# Fuzzy cognitive maps for municipal governance improvement

**DOI:** 10.1371/journal.pone.0294962

**Published:** 2024-02-29

**Authors:** Lenin Parreño, Federico Pablo-Martí

**Affiliations:** 1 Pontifical Catholic University of Ecuador PUCE, Quito, Ecuador; 2 SCCS Research Group, University of Alcala, Alcalá de Henares, Spain; FURG: Universidade Federal do Rio Grande, BRAZIL

## Abstract

This paper applies Fuzzy Cognitive Maps (FCMs) to understand the diverse behavior of municipal governments in Ecuador to find common elements that influence the well-being of citizens in the short and long term. Information gathering was conducted in two stages: in the first one, a group of 16 national experts was consulted to develop the initial FCM; in the second stage, local experts from 220 municipalities were interviewed to collect information on the general validity of initial FCMs and specific values given to concepts and relationships in their municipalities. Results show the importance of certain concepts for long-term municipal performance, such as the need for a competitive entrepreneurial sector, improving human resources in the municipality, and, particularly, having a competent mayor with leadership skills and a forward-looking vision that enables the development of municipal projects required to reach an efficient and equitable coverage of goods and services throughout the city. Through the application of genetic algorithms, the FCM was calibrated to ascertain the long-term dynamics of municipal development and the optimal values of the concepts that would optimize the attainment of the set objectives. The derived outcomes suggest the desirability of the maintenance of, in principle, unwanted structures like financial transfers from the central government and the need to exploit natural resources to attain urban development.

## Introduction

Complexity theory surfaces during the last three decades due to interactions between disciplines as diverse as Physics, Biology, or Economics, to model heterogeneous phenomena labeled complex systems [1].

These systems share some properties [2, 3]. They are open systems where interactions between elements are unclear since relationships, in many cases, are nonlinear and evolve and adapt over time. Elements that form a complex system cannot be understood as isolated entities when considering the relationships and the environment from which they develop. Thus, if viewed separately, behaviors that may be granted little significance become relevant when they interconnect within a network that seeks to self-organize [4]. This self-organizing process breeds new structures and behaviors situated within their environment.

Another essential characteristic comprises those past events which influence complex systems in a significant way. Even then, the behavior of such systems is difficult to predict accurately, given their ability to adapt and learn from interactions with other complex systems.

Complex reflexive systems (CRS) are a particular group of complex systems characterized by the fact that the types of networks that form them are more critical than their constitutive elements [5]. The relationships between elements are a fundamental aspect of complex systems because they play a more significant role in defining the latter than the elements that comprise it.

Independent of their geographical jurisdiction, public administration organizations can be considered a CRS, which implies that the only way to manage the instability of those events occurring is to rely on an adequate level of flexibility. This requires providing organizations with the necessary tools to establish the simplifying rationality that will allow for environmental control, creating a new order [6].

Organizations such as municipalities are a product of nonlinear interactions and relations between their agents, generating stable or unstable behaviors over time (chaotic zone) [7].

In this sense, city mayors do not strive for permanent balance over time but seek ways to innovate and change adaptively [8], to improve citizens’ quality of life and keep themselves in office.

Public officials, for their part, also respond to their own emotions and ambitions, which are not always aligned with the well-being of the general population. This group seeks to understand power dynamics, persuade others, and follow directives; it systematically thinks and observes interacting with external actors and elements [9].

Managers, planners, and other critical stakeholders in the local public policy field must develop their strategies for territorial development in complex environments characterized by incomplete information. Their main objectives should be to increase certainty and trust among economic stakeholders [10], stabilize chaotic systems to the best of their ability, impose limits to certain fluctuations, and create desirable institutional structures [11]. Nonetheless, they fail in this challenge because general answers are sought for complex problems using specialized models [12].

One way of establishing effective institutional structures is through natural self-organizing mechanisms, assuming that social structures are built over time and that their cause-and-effect relations prove far more complex than simply determining the association between two variables [13]. Among such mechanisms are Fuzzy Cognitive Maps (FCMs).

FCMs have been used more frequently in the hard sciences to resolve flux and energy control issues and industrial, technological, informational, and business processes [14]. However, recently, they have also found applications in fields as diverse as medicine, climate change, institutional analysis, or mapping of key stakeholders [15–17].

Regarding the management of territories such as cities and regions, the literature shows the use of FCMs in areas that include: urban systems [18], water [19] and waste [20, 21], management systems, urban planning [22] (Habib and Shokoohi, 2009, management systems, urban planning [22, 23] (Habib and Shokoohi, 2009, and land usage [24], economic, social and environmental assessments [15], and traffic control [18]. In this last field, Olazabal and Pascual [25] demonstrate the importance of FCMs for city management as they facilitate confronting problems that cannot be analyzed from a positivist and theoretical viewpoint; on the contrary, FCMs contribute towards the analysis of dynamic and unbalanced systems, as occurs in urban settings [25].

In a city, defining a network works as a mechanism for observing the density and centrality of the components that form an FCM. Thus, the connection between concepts and causal relationships, the modes of interaction between main actors, and their forms of adaptation and learning are elements that city mayors should analyze daily in addition to processing them systematically to develop new knowledge that facilitates future public policy design and implementation.

Each municipality involves a system and network that transforms itself over time; this implies that each city must develop its own FCM based upon the structure of its components and heterogeneous behavior adjusted to specific realities and settings.

This work applies FCM to understand the behavior of various Ecuadorian municipalities and establish the common elements that affect citizens’ well-being over the short and long run. Answers are sought for the following three key questions:

- Which key concepts describe municipal performance in Ecuador as measured through the equitable and efficient production and delivery of goods and services?
- Which are the main restrictions that municipalities in Ecuador face in improving their performance regarding city management?
- Which institutional variables regarding public policy design and action plans could have a greater short-term and long-term influence on municipal performance?

## Material and methods

The Fuzzy Cognitive Map (FCM) concept was initially developed by Axelrod [26] to represent social scientific knowledge. The FCM is a simple graph formed by "nodes" and "arcs." Nodes represent relevant concepts of a given domain (*N*_1_, *N*_2_, *N*_3_,…*N*_*n*_) while causal relationships between them are represented by connecting arcs.

Each arc is associated to a (+) or (−) sign. A positive arc that connects node *N*_1_ and node *N*_2_ indicates a positive influence of *N*_1_ over *N*_2_, which implies that higher values in node *N*_1_ will have a positive effect on *N*_2_. The inverse situation occurs when the sign is negative and not limited to binary values, but open to the insertion of continuous numerical values. This makes it feasible to use a range of values from −1 to +1 [27].

Causal relationships between nodes are associated with a number or "weight" that determines the degree to which the dependent node relates to the incoming node. The weight (ei,j) between two nodes is usually normalized into the interval [–1,1], where -1 represents an entirely negative influence or effect, 0 means there is no causal relationship, and +1 stands for a wholly positive impact [28].

It should be noted that an FCM is a simplified representation of a problem described by a set of concepts (nodes) and their respective causal relationships (arcs), which are associated with a numeric value denoting the "weight" between nodes. These causal-effect relationships are organized into a square matrix known as a "connection matrix," where each element indicates the weight of a specific causal relationship in the FCM.

The formal description of an FCM is presented next: $N=\{N_{1},N_{2},\ldots N_{12}\},\;is\;the\;set\;of\;nodes$

$$E:\;(N_{i},N_{j}),\to e_{ij}$$

*E* represents a function of *N x N* whose values (*K*) fall within the interval [−1,1], associating *eij* to a pair of concepts (*N*_*i*_, *N*_*j*_). It is helpful to remember that *eij* represents the direct weight (arc) between node *N*_*i*_ and node *N*_*j*_, as long as *i*≠*j* or *eij* = 0, if *i* = *j*. Thus, the connection matrix is modeled in the following way:

$$E(N\;x\;N)=(e_{ij})\epsilon \;K^{nxn}$$

Connections between nodes can be expressed as:

$$C:\;N_{i}\to C_{i}$$

*C* represents a function where each concept *Ni* is associated to the sequence and degree of activation, as shown below:

$$t\;\epsilon \;N,C_{i}(t)\;\epsilon \;L,$$

*L* = [0,1] is the activation vector that specifies the initial concept values introduced at each node, and *C*(*t*) *ϵ L* is the state vector at a given number of iterations *t*.

The transformation function, which includes recurring relationships on *t*≥0 between *C* (*t*+1) and *t*, is represented as:

f:R→L

$$\forall i\in \{1,\ldots ,n\},\;C_{i}(t+1)=f\left(\sum_{\begin{array}{c} i=1\\ j\neq 1 \end{array}}^{n}e_{ij}C_{j}(t)\right)$$

FCM has four key elements *F* = (*N*, *E*, *C*, *f*); however, function *f* is used to perform simulations in dynamic systems. Simulations start with the "state vector," which specifies initial values for all concepts (nodes) in a particular number of iterations "t."

The value for a node is calculated through the iteration of values for other nodes that exert influence (+/−) on the given node via cause-effect relationships determined in the FCM.

The aim of the transformation function is to confine the weighted sum to a specific interval which is usually set to [0,1]. There are several transformation functions, including bivalent, trivalent, etc. This paper uses a logistic function, as shown next:

$$f(x)=\frac{1}{1+e^{-C_{x}}}$$

Parameter C is used to determine the shape of the function that serves to implement several simulation scenarios [29]. When simulations are discrete (bivalent, trivalent, etc.), they lead to a fixed value known as a "fixed point attractor" or "hidden pattern." Otherwise, the function will continue cycling between fixed values of the state vector, a behavior known as a "limit cycle." When using a continuous function, such as the logistic one, the hidden pattern and the limit cycle give place to the "chaotic attractor." This term is used when the state vector produces different values for successive iterations or cycles.

The FCM results from a sequence of state vectors and represents the possible scenarios for a system based on iterations starting from initial conditions.

### Learning

Stach *et al*. [27] explain how in FCMs, several algorithms are developed based on what is known as "learning processes." The most used ones are the Hebbian Algorithm and the Genetic Algorithm. However, up to now, there is no methodological agreement on how to develop an FCM [30].

Kosko [31] proposed the Differential Hebbian Learning Law (DHL) for application to FCMs via the correlation of changes in concepts or nodes, as shown next:

$${\dot{e}}_{ij}=-e_{ij}+{\dot{C}}_{i}{\dot{C}}_{j}$$

Where ${\dot{e}}_{ij}$ represents the change of weight between concepts *i*_*th*_ and *j*_*th*_, while *e*_*ij*_ is the current weight value, and the term ${\dot{C}}_{i}{\dot{C}}_{j}$ stands for changes in values *i*_*th*_ and *j*_*th*_, respectively.

Learning processes in genetic algorithms are gradual dynamics that iteratively update all the weights of causal relationships until the desired connection matrix is found, based on the following conditions:

$$e_{ij}(t+1)=\left\{ \begin{array}{l} e_{ij}(t)+c_{t}[\Delta C_{i}\Delta C_{j-}e_{ij}(t)]\;si\Delta C_{i}\neq 0\\ e_{ij}(t)\;si\Delta C_{i}=0 \end{array}\right.$$

Where *e*_*ij*_ is the weight of the causal relationship between *C*_*i*_ and *C*_*j*_; Δ*C*_*i*_ represents the change in the activation value for the concept *C*_*i*_; *t* is the iteration number and *c*_*t*_ is the learning coefficient. The aim is to find values for the connection matrix via predetermined input and output values for state vectors and an iterative simulation process.

Golberg [32] explains that GA were originally based on natural genetics and, as pointed out by Deb [33], they currently have various applications in optimization processes and search activities for minimizing resources, given their broad applicability, ease of use and the integration of a global perspective.

Learning systems in FCMs use the Real Coded Genetic Algorithm (RCGA), which incorporates the chromosome concept understood as a group of floating numeric values. This algorithm performs a linear transformation for each variable of the solution to decode it into the desired interval. The main components of the RCGA are summarized next.

The RCGA defines each chromosome as a floating-point vector whose length is determined by the number of variables in the problem. Each element in the vector is called a "gene;" for a learning FCM, each chromosome consists of N(N-1) which consists of genes that are floating numbers in the interval [–1,1] defined as follows:

$$\tilde{E}={\left[e_{12},e_{13},\ldots ,e_{1N},e_{21},e_{23},\ldots ,e_{2N}\ldots \ldots \ldots e_{NN-1}\right]}^{T}$$

Where e_ij_ is the weight for each causal relationship (arc), from the concept "i" to concept "j." Each chromosome must once again be decoded within the FCM; this involves copying the weight value for each chromosome into the corresponding cell of the connection matrix. It is worth mentioning that in the RCGA, the number of chromosomes in a population is constant for each generation, and the "population size" must be specified as one of the parameters for the map.

The simulation of the state vector C(t+1) in the FCM depends on the iteration that precedes it; this meaning that the system for the map achieves a state that has already been reached in an immediately preceding iteration. Therefore, explained behavior considers its history. The result for this process is termed "limit cycle," where the input value has a *K* range, but the limit cycle or fixed-point attractor occurs when the condition *L*<*K* is met in the *L*_*th*_ iteration. In this way, the input value can be used to learn but is limited (truncated) to the first *L* iterations.

The process produces two vectors: *C*(*t*) as the initial vector and *C*(*t*+1) as the vector that collects answers from the system, generating *K*−1 pairs. A higher *K* value provides more information on the system’s behavior. Thus, the fitness function is calculated by computing for each chromosome the difference of the system response generated from using a candidate from the FCM, and another one that comes directly from input values. The system response of the FCM candidate is computed by decoding the chromosome in the FCM through the simulation and iteration of the initial state vector taken as the first input value. This difference is computed across all *K*−1 pairs formed by the system response vector and the initial state vector.

The fitness function is measured in the following way:

$$Fitness\;function=h(Error\_Lp)=h(\emptyset \sum_{t=1}^{K-1}\sum_{n=1}^{N}{\left|C_{n}(t)-{\tilde{C}}_{n}(t)\right|}^{p})$$

Where *h* is an auxiliary function.

The introduction of this function serves two primary purposes:

- It promotes improved individual behavior by minimizing the cumulative error.
- It rectifies chromosomes through non-linearity, thereby facilitating their approximation towards the targeted solution.

The function is articulated as h(x) = 1/(ax+1), wherein the parameter ’a’ is experimentally determined. Furthermore, the fitness function is normalized within the range [0,1].

The ideal value is attained when the state vector aligns with the vector comprising the input values.

C(t)=[C1(t),C2(t),…,Cn(t)] denote the system response of the model for each *C*(*t*−1) initial vector, and $\tilde{C}(t)=[{\tilde{C}}_{1}(t),{\tilde{C}}_{2}(t),\ldots ,{\tilde{C}}_{n}(t)]$ is the system response of the candidate vector of the FCM for each C(t-1) initial vector; *p* = ∞ and ∅ is the parameter used to normalize the error, equal to 1 / (K-1).

Iterations stop in two scenarios: a) when the learning process is considered successful, i. e., the state vector resulting from the simulation is equal or close to the input value based on the fitness function, and b) when the learning process is unsuccessful; this happens when despite a maximum number of iterations for the generations, it proves impossible to achieve a good fit.

To reach this goal, it is necessary to perform a recombination using crossover operations with one or several crossover points. Nocedal et al. [34], Herrera et al. [35] and Michalewicz [36] suggest some operators apply an RCGA. These include, for example, random mutation, non-uniform mutation and Mühlenbei mutation, among others. This recombination involves simultaneous operations. The most widely used selection strategies are the roulette wheel selection and the tournament selection [27].

To summarize, an FCM is developed by identifying key concepts, causal relationships and weights based on expert knowledge of a situation or problem one wishes to understand. At a later stage, a genetic algorithm is used to train initial weights in the state matrix. Through an iterative process and the use of genetic algorithms, new weights and relationships between concepts are found; this involves an evaluation, selection and recombination system, crossover points and mutation. After this iterative process, the FCM will have a new weight matrix and new concept values, allowing an assessment of their distance from the initial values introduced into the represented system; this, in turn, allows identifying whether the FCM achieves a learning process or not.

## Theory/Calculation

The development and analysis of the FCM was conducted in four stages:

**Stage 1:** Developing the initial FCM based on the definition of key concepts that determine municipal performance along with the main interrelationships between them. This was done based on information gathered in 2016 through four rounds of meetings with sixteen national experts who had worked in important posts related to planning and cooperation activities, academic research, and professional services for canton-level organizations. This group also included a former mayor and several officials from the Ministry of Finance who were previously in charge of evaluating municipal finances, debt capacity, and allocation of transfers from the central government to sub-national or local governments.

**Stage 2:** Establishing FCM values for each of the 220 municipalities in Ecuador through a questionnaire with thirty-two questions which identify the intensity of the relationships between concepts. The questionnaire, designed in conjunction with experts, was administered to municipal specialists identified for each of the 220 cities.

**Stage 3:** Modeling and simulating scenarios for different types of municipalities. We assume that the relationships between concepts identified by experts and displayed in FCMs are basically correct, particularly concept values, although their internal coherence is only partial. The iterative application of the Hebbian learning process leads to a model with stable relationships in the long term.

**Stage 4:** Using genetic algorithms to develop a relationships structure between concepts that stabilize optimum values over the long term.

These four stages brought about an analysis and intervention framework that Ecuadorian municipalities can use to improve their financial and social performance.

### Developing the initial FCM

There are two approaches for developing FCMs: computational and manual. The computational approach automatically chooses concepts and relationships based on the following criteria: efficiency related to complexity and efficacy in achieving results.

In contrast, manual FCMs are developed based on the knowledge of one or several experts concerning a specific reality (domain model). This usually causes manual FCMs to be more complex in structure and with lesser predictive capacity than computational FCMs; on the positive side, they tend to display fewer spurious relationships and are more stable over time, which makes them more suitable in order to obtain an in-depth knowledge of the internal structure of complex systems [37]. Based on the foregoing reasons, in this paper, we opt for a manual approach to create the initial FCM.

The process we followed is structured into four stages:

1. Identifying key concepts and nodes agreed upon with experts through several discussion rounds. In this specific case, the structured communication Delphi method was used for this purpose [38, 39].
2. Defining causal relationships between nodes or concepts using the same procedure employed in the first stage. When using the Delphi technique, we used the methodology proposed by Stylios and Groumpos [40], that recommends establishing first the sign of cause-effect relationships (positive, negative or none).
3. Establishing the intensity of identified causal relationships in fuzzy terms, that is, through categories such as very high, high, medium, low, very low, taking as a reference the previously determined influence sign between two nodes.
4. To confirm the criteria of experts and to achieve more coherence and stability for results, a Hebbian learning process was applied to the initial and final values of the state vector, which in turn served to find the value of weights for each causal relationship.

To accumulate the expert knowledge necessary for the composition of the Fuzzy Cognitive Map (FCM), a Delphi methodology was employed, featuring 16 local government specialists from diverse knowledge spheres associated with municipalities in Ecuador. This assembly incorporated technical professionals experienced in planning processes, collaborative activities, scholarly research, and professional services rendered to cantonal entities. Additionally, insights were sought from a former mayor and functionaries of the Ministry of Finance who have overseen the assessment of municipal finances, debt capacity, and the allocation of fiscal transfers from the Central Government to Sub-national Governments.

Selection of experts was guided by key criteria such as: a) tenure in the field of municipal issues, b) comprehensive understanding and expertise in national municipal affairs, c) professional, academic or administrative roles that facilitated their association with municipalities, d) accessibility and time availability to participate in bilateral and group discussions with other experts, and e) capacity to articulately present and debate their experiences in the municipal domain. The list of experts is included in the annex.

The preliminary round solicited responses to an open-ended question concerning the primary variables influencing municipal efficiency and equity in the production and delivery of municipal goods to city inhabitants.

In the ensuing round, all variables presented by the experts were consolidated and experts were queried regarding the ten most significant attributes in municipal administration. This stage facilitated a consensus regarding the critical factors affecting municipal performance. Despite the restriction to ten variables, this expert consultation process resulted in the identification of 12 key concepts or nodes for the construction of the Dynamic Cognitive Map (DCM).

The third round revisited the variables identified and agreed upon in the second stage, asking the experts to identify the nature of influence (positive or negative) among each of these agreed variables. This phase facilitated the construction and identification of causal relationships or arcs between each of the concepts, culminating in a total of 31 arcs, and yielding the initial configuration of the DCM of Municipalities in Ecuador.

The fourth round relied upon the causal relationships determined in the previous phase and posed questions based on the questionnaire appended, aimed at gauging the qualitative weight of the concepts (nodes) as well as the causal relationships within each of the previously identified arcs. This survey addressed two fundamental elements: the first pertains to understanding the average and current behaviors of the nation’s municipalities; the second involves identifying the desired value of the weights of the concepts and the cause-effect relationships.

Following the acquisition of the initial values of the municipal DCM from the expert standpoint, the subsequent step involved administering the prior survey to officials and professionals with an extensive understanding of municipal performance across the nation’s 220 cities. The objective of this was to examine the institutional level municipal heterogeneity identified in the third chapter and to discern potential indications of learning among these subnational entities based on the application of genetic algorithms.

It is worth mentioning the latter four stages served as the basis to create the survey that gathered information on the weight of concepts and the value of cause-effect relationships at the municipal level. Respondents were able to choose their answers from a scale offering five options: 1 (very high), 0,75 (high), 0,5 (medium), 0,25 (low), and 0,10 (very low). These options were applied to the twelve concepts and thirty-two cause-effect relationships identified in the rounds of meetings held with experts.

**Fig 1:**
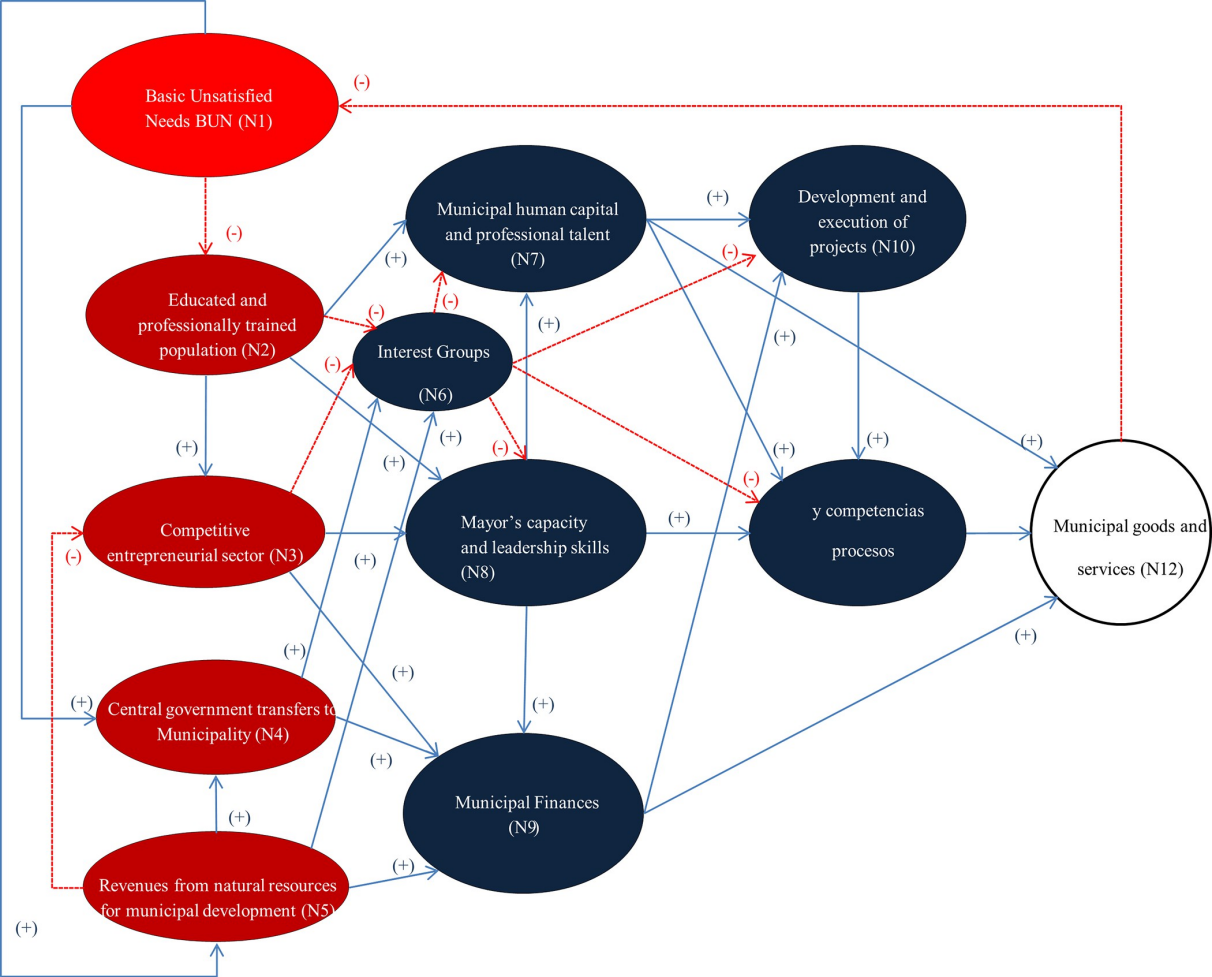
Initial FCM that explains municipal performance in Ecuador.

Fig 1 shows the initial Fuzzy Cognitive Map developed to understand the performance of Ecuadorian municipalities. This FCM derives from the knowledge of municipal experts and includes twelve nodes (*N* = 12) and thirty-one connections (*Cij*), represented by an *N x N* (12*x*12) connection matrix.

The FCM has as its main input the *Unsatisfied Basic Needs* (UBN) of the canton (N1), which negatively affects the growth of the educated and professionally trained population (N2), who could, however, be positively compensated through the emergence and development of a competitive entrepreneurial sector (N3). If UBN increase, the central |government is forced to make greater capital transfers to the municipality (N4), and the population may have more incentives to use natural resources and generate income that finances municipal development (N5).

A greater number of educated individuals in the city will negatively impact the emergence of rent-seeking groups within the municipality (N6) and will have a positive effect on the municipal human capital (N7); this educated population will also have greater possibilities of positively impacting the mayor’s leadership and competence, which are considered basic skills for city development (N8).

Municipal finances will improve (N9) and prosper if the population is increasingly educated and has a professional degree. This concept will also improve if the mayor’s administration ensures adequate municipal income, expenditures, and debt management. Other concepts that enhance the municipal financial flux include transfers from the central government and raising revenues from natural resources.

The design and execution of municipal projects (N10) will be enhanced insofar as the mayor’s office incorporates professional and technical human capital and financial resources to achieve feasible public investment within the city; however, it will be negatively affected by the appearance of rent-seeking groups.

The development of internal municipal processes and competencies (N11) will positively depend on the mayor’s skills and vision for the future, along with the availability of municipal human resources and skills for designing and executing city development projects.

Finally, the efficient and equitable production and provision of public municipal goods and services (N12), depends on human resources, process development within the municipal government, and municipal finances. Therefore, if more municipal goods and services are delivered to the population efficiently and equitably, this would reduce UBN among the city’s population (N1).

## Results

### The ideal municipality and the actual situation

Meetings with municipal experts allowed us to identify nodes (concepts) decisive for municipal performance and led to a description of the desirable municipality in Ecuador.

Using the same format previously described for the survey, the first column of Table 1 shows experts’ criteria regarding an "ideal or desirable municipality," which should possess a low level of UBN (*N*_1_ = 0,25), a large educated and professionally trained population (*N*_2_ = 0,75), a highly competitive entrepreneurial sector (*N*_3_ = 0,75), and low dependence of the municipal budget on transfers from the central government (*N*_4_ = 0,25).

**Table 1:** Desirable and real concept values in municipalities, according to experts.

N_i_	FCM Concepts	N* (ideal)	N_i_	Gap (N*- N_i_)
1	Basic Unsatisfied Needs of the population in the canton	0.25	0.58	0.33
2	Presence or development of an educated and professionally trained population	0.75	0.48	-0.27
3	Presence or development of a competitive entrepreneurial sector	0.50	0.41	-0.09
4	Importance of transfers from the central government to the municipality	0.50	0.71	0.21
5	Need for using natural resources to obtain revenues that aid municipal development	0.10	0.47	0.37
6	Presence of rent-seeking and other interest groups	0.25	0.51	0.26
7	Current state of the municipal human capital and professional talent	0.75	0.58	-0.17
8	Capacity and leadership skills of the mayor to guide city development	0.75	0.81	0.06
9	Importance of municipal finances for urban development	0.75	0.70	-0.05
10	Skills to design and execute municipal projects	0.75	0.59	-0.16
11	Development or current state of municipal processes and competencies	0.75	0.56	-0.19
12	Level of efficient and equitable coverage of municipal goods and services	0.75	0.52	-0.23

Based on this, other desirable criteria include a slight need for the use of natural resources to sustain municipal finances (*N*_5_ = 0,10), scarce opportunistic and rent-seeking groups (*N*_6_ = 0,10), and a high level of human capital and talent within the professional and technical municipal team (*N*_7_ = 0,75).

The previous assertion is compatible with the reliance on a mayor with a very high level of competence, leadership, and ability to visualize and plan for city development (*N*_8_ = 1), a significant influence of municipal finances on city development (*N*_9_ = 0,75), a high municipal capacity to develop and execute public investment projects (*N*_10_ = 0,75), as well as highly developed municipal processes and competencies (*N*_11_ = 0,75).

The values of the concepts previously described will allow for high coverage of public municipal goods and services produced and delivered efficiently and equitably (*N*_12_ = 0,75).

The second column in Table 1 shows experts’ assessment regarding what occurs in municipalities. Thus, for example, this group of municipal specialists determined that gaps are significant for almost every node or concept of the FCM; however, what proves interesting is the difference in gaps for certain concepts, such as the high dependency on transfers from the central government, the appearance of interest rent-seeking groups, and the absence of municipal processes.

## Discussion

To determine the coherence of values assigned by the expert panel and those gathered through the survey in municipalities, we compared the medium values for concepts attained through both methods (Table 2). The results verified the superior knowledge of national experts on Ecuadorian municipalities and the reliability of the information collected through the survey administered in municipalities. The only remarkable difference is the excessively high value given by interviewees to mayors’ competency, vision, and leadership versus the value for the same concept given by experts, which is probably related to the fact municipal government employees will tend to elude criticism of their superiors for fear of retaliation.

**Table 2:** Comparison of values given to nodes by experts and actual national values.

N_i_	FCM Concepts	N* (expert)	N_i_ nat. average	Gap (N_exp_- N_i_)
1	Basic Unsatisfied Needs of the population in the canton	0.57	0.61	-0.04
2	Presence or development of an educated and professionally trained population	0.42	0.47	-0.05
3	Presence or development of a competitive entrepreneurial sector	0.40	0.34	0.05
4	Importance of transfers from the central government to the municipality	0.78	0.79	-0.01
5	Need for using natural resources to obtain revenues that aid municipal development	0.59	0.49	0.09
6	Presence of rent-seeking and other interest groups	0.60	0.52	0.08
7	Current state of the municipal human capital and professional talent	0.42	0.47	-0.05
8	Capacity and leadership skills of the mayor to guide city development	0.64	0.81	-0.17
9	Importance of municipal finances for urban development	0.73	0.70	0.03
10	Skills to design and execute municipal projects	0.39	0.42	-0.04
11	Development or current state of municipal processes and competencies	0.34	0.40	-0.06
12	Level of efficient and equitable coverage of municipal goods and services	0.48	0.46	0.03

Weights for the cause-effect relationships between nodes in the connection matrix in Table 3, developed after processing the survey administered to the universe of municipalities, show that most causal relationships are between 0,4 and 0,5. This implies a medium effect or degree of influence, except for the "high" positive effects related to mayors’ leadership, the development of human resources and talent within the municipality, the improvement of municipal finances and the implementation of municipal processes.

**Table 3:** Connection matrix: Average values for country-wide cause-effect relationships.

	C1	C2	C3	C4	C5	C6	C7	C8	C9	C10	C11	C12
C1	0.00	-0.57	0.00	0.57	0.49	0.00	0.00	0.00	0.00	0.00	0.00	0.00
C2	0.00	0.00	0.47	0.00	0.00	-0.49	0.58	0.61	0.00	0.00	0.00	0.00
C3	0.00	0.00	0.00	0.00	0.00	-0.43	0.00	0.53	0.50	0.00	0.00	0.00
C4	0.00	0.00	0.00	0.00	0.00	0.47	0.00	0.00	0.63	0.00	0.00	0.00
C5	0.00	0.00	-0.43	0.46	0.00	0.43	0.00	0.00	0.49	0.00	0.00	0.00
C6	0.00	0.00	0.00	0.00	0.00	0.00	-0.44	-0.46	0.00	-0.45	-0.47	0.00
C7	0.00	0.00	0.00	0.00	0.00	0.00	0.00	0.00	0.00	0.64	0.63	0.64
C8	0.00	0.00	0.00	0.00	0.00	0.00	0.69	0.00	0.71	0.00	0.71	0.00
C9	0.00	0.00	0.00	0.00	0.00	0.00	0.00	0.00	0.00	0.65	0.00	0.62
C10	0.00	0.00	0.00	0.00	0.00	0.00	0.00	0.00	0.00	0.00	0.63	0.00
C11	0.00	0.00	0.00	0.00	0.00	0.00	0.00	0.00	0.00	0.00	0.00	0.60
C12	-0.57	0.00	0.00	0.00	0.00	0.00	0.00	0.00	0.00	0.00	0.00	0.00

These results are consistent with the previous values assigned by municipal officials who consider that mayors, on average, are highly capable and have good leadership skills to conduct the municipal government and improve the quality of life in the canton.

### Heterogeneous dynamics in municipalities

This section shows changes generated in local development plans and, therefore, in FCMs, due to variations in initial conditions.

A basic scenario depicting the average behavior of municipalities is analyzed and contrasted with the conduct of fifteen associations of municipalities that share specific characteristics considered relevant in previous descriptive analyses.

The analysis begins with the identification of initial state vectors ($N_{i}^{0}$) that indicate the initial value for the twelve concepts and nodes represented in the FCM for the basic scenario (nationwide) and the other fifteen scenarios, as shown in Table 4.

**Table 4:** Initial state vectors.

		National level	High EBM	High ISFM	Urban pop.	Rural pop.	Agricult. Act.	Manuf. Ind.	Profess. Act.	Priv. Sect. Employ.	Higher Educat.	Financial Dep.	Univeristies	Seniority	Distance	Altitude	Temperature
1	Basic Unsatisfied Needs of the population in the canton	0.6	0.5	0.6	0.6	0.6	0.6	0.6	0.6	0.6	0.6	0.7	0.6	0.6	0.6	0.6	0.6
2	Presence or development of an educated and professionally trained population	0.5	0.6	0.5	0.5	0.5	0.5	0.5	0.5	0.5	0.57	0.5	0.5	0.5	0.5	0.5	0.5
3	Presence or development of a competitive entrepreneurial sector	0.3	0.4	0.5	0.5	0.3	0.3	0.4	0.4	0.5	0.4	0.5	0.4	0.3	0.3	0.4	0.3
4	Importance of transfers from the central government to the municipality	0.8	0.7	0.8	0.7	0.8	0.8	0.7	0.8	0.8	0.8	0.7	0.8	0.8	0.8	0.8	0.8
5	Need for using natural resources to obtain revenues that aid municipal development	0.5	0.5	0.5	0.6	0.4	0.5	0.5	0.5	0.5	0.5	0.6	0.6	0.5	0.5	0.5	0.5
6	Presence of rent-seeking and other interest groups	0.5	0.5	0.54	0.6	0.5	0.5	0.5	0.5	0.6	0.5	0.6	0.5	0.5	0.5	0.5	0.5
7	Current state of the municipal human capital and professional talent	0.5	0.6	0.6	0.6	0.4	0.5	0.5	0.5	0.5	0.6	0.6	0.6	0.5	0.5	0.5	0.5
8	Capacity and leadership skills of the mayor to guide city development	0.8	0.7	0.8	0.8	0.8	0.8	0.8	0.8	0.8	0.8	0.9	0.8	0.8	0.8	0.8	0.8
9	Importance of municipal finances for urban development	0.7	0.7	0.7	0.7	0.7	0.7	0.7	0.7	0.8	0.7	0.7	0.7	0.7	0.7	0.7	0.7
10	Skills in the design and execution of municipal projects	0.4	0.5	0.5	0.5	0.4	0.4	0.5	0.5	0.5	0.5	0.5	0.5	0.5	0.4	0.4	0.4
11	Development or current state of municipal processes and competencies	0.4	0.5	0.4	0.4	0.4	0.4	0.5	0.4	0.4	0.4	0.4	0.4	0.4	0.4	0.4	0.4
12	Level of efficient and equitable coverage of municipal goods and services	0.5	0.6	0.6	0.5	0.4	0.4	0.5	0.5	0.5	0.5	0.3	0.6	0.5	0.4	0.4	0.5

To better understand these vectors, it is necessary to compare the situation of an ideal municipality, the experts’ criteria, and average national behavior.

Table 5 displays the percentage gap between the actual and the desirable behavior of a municipality. A first finding is that in each scenario, average gaps are above 60%; among this group, the most notorious gaps are the lack of a competitive entrepreneurial sector, the dependence of municipal finances on central government transfers, the need for greater use of natural resources to improve municipal cash flow, the presence of interest groups, and the lack of process development within the municipality.

**Table 5:** Percentage gap between initial concepts and the ideal situation.

		National level	High EBM	High ISFM	Urban pop.	Rural pop.	Agricult. Act.	Manuf. Ind.	Profess. Act.	Priv. Sect. Employ.	Higher Educat.	Financial Dep.	Univeristies	Seniority	Distance	Altitude	Temperature
1	Basic Unsatisfied Needs of the population in the canton	59	48	57	60	61	60	58	56	56	58	62	57	58	59	56	58
2	Presence or development of an educated and professionally trained population	58	29	37	49	58	59	46	47	51	32	41	35	45	56	61	54
3	Presence or development of a competitive entrepreneurial sector	117	65	61	52	139	123	67	86	56	69	41	69	112	124	111	128
4	Importance of transfers from the central government to the municipality	68	65	68	65	69	69	66	68	68	67	65	68	69	69	68	68
5	Need for using natural resources to obtain revenues that aid municipal development	80	80	79	82	77	80	80	81	81	80	83	83	80	80	80	80
6	Presence of rent-seeking and other interest groups	81	81	81	84	79	81	81	82	82	81	83	80	80	81	81	81
7	Current state of the municipal human capital and professional talent	59	26	23	35	69	61	40	40	37	29	20	32	45	59	64	57
8	Capacity and leadership skills of the mayor to guide city development	23	33	30	32	20	23	24	31	27	27	14	32	19	24	21	26
9	Importance of municipal finances for urban development	0	0	0	0	0	0	0	0	0	0	0	0	0	0	0	0
10	Skills in the design and execution of municipal projects	77	36	46	48	89	81	49	61	57	50	50	50	60	82	80	75
11	Development or current state of municipal processes and competencies	87	54	74	86	88	90	64	82	71	72	71	74	83	91	85	91
12	Level of efficient and equitable coverage of municipal goods and services	612	34	28	41	80	67	42	55	41	43	41	33	58	65	68	60
	*Average gap in node values in relation to the ideal situation*	59	44	46	51	61	60	49	54	50	48	45	49	54	60	59	59

The survey information also reveals no gap concerning the importance of municipal finances for city development. The second-lowest gap involves the need for a mayor with a forward-looking vision and leadership skills.

The basic national scenario shows a gap of 59,14% concerning the ideal situation. It is worth noticing this gap is reduced in municipalities characterized by a high EBM (Spanish acronym for Basic Municipal Evaluation), high ISFM (Spanish acronym for Family Satisfaction Index in the Municipality), more urban population, higher production of manufactured goods, private sector employment, increasing population with access to higher education, and less dependency on transfers from the central government. However, the gap increases when the context for the development of municipalities includes more participation of the rural population, more sophisticated agricultural activities, and longer distances from bigger cities. Cities with an average temperature above 20 degrees Celsius and an average altitude below 1500 m.a.s.l, display similar behavior to the national average, which seems coherent given the number of municipalities they represent across the country.

Once the initial state vector is established, the next step is to calculate the new values for nodes or concepts through an iterative process that involves multiplying the vector state by the connection matrix. This is made possible by using an activation function to observe the monotonic behavior of node values normalized into a range of [0,1]. To do this, we followed the recommendation of Bueno & Salmeron [41], who suggest the use of a sigmoid function, as shown below:

$$C_{i}^{t+1}=f\left(C_{i}^{t}+\sum_{j\neq 1}w_{ij}.C_{j}^{t}\right)$$

$C_{i}^{t}$ is the state vector value in moment *t*; *w*_*ij*_ is the weight and influence of node *j* on node *i*, and *f*(.) is the sigmoid activation function represented as follows:

$$C_{i}^{t+1}=\frac{1}{1+e^{-\lambda (C_{i}^{t}+\sum_{j\neq 1}w_{ij}.C_{j}^{t})}}$$

After the corresponding process and several iterations, the system described in the FCM reaches the "hidden node value" or "fixed-point attractor," meaning the node value stabilizes.

Table 6 shows a decrease (-) or increase (+) when comparing gaps for new concept values after using the activation function and gaps shown in Table 5; this helps pedagogically depict the iteration results and the convergence state of the base scenario and the 15 variations.

**Table 6:** Variation rate of gaps for new concepts concerning gaps for initial state vectors.

		National level	High EBM	High ISFM	Urban pop.	Rural pop.	Agricult. Act.	Manuf. Ind.	Profess. Act.	Priv. Sect. Employ.	Higher Educat.	Financial Dep.	Univeristies	Seniority	Distance	Altitude	Temperature
1	Basic Unsatisfied Needs of the population in the canton	-31	4	-29	-39	-34	-32	-29	-25	-27	-33	-45	-23	-26	-31	-34	-25
2	Presence or development of an educated and professionally trained population	-29	14	-6	-19	-27	-30	-17	-18	-22	7	-11	2	-16	-30	-31	-26
3	Presence or development of a competitive entrepreneurial sector	-66	-55	-51	-42	-70	-67	-54	-60	-47	-53	-28	-54	-66	-67	-65	-68
4	Importance of transfers from the central government to the municipality	-9	5	-7	8	-12	-10	0	-6	-6	-3	8	-4	-10	-11	-8	-9
5	Need for using natural resources to obtain revenues that aid municipal development	46	46	50	23	66	46	48	32	36	47	22	19	46	47	47	45
6	Presence of rent-seeking and other interest groups	29	25	24	8	41	30	30	20	19	24	17	40	35	32	27	30
7	Current state of the municipal human capital and professional talent	-93	-91	-86	-84	-95	-94	-91	-89	-87	-83	-73	-76	-93	-94	-93	-95
8	Capacity and leadership skills of the mayor to guide city development	59	13	27	20	78	61	51	26	37	41	131	27	86	54	70	53
9	Importance of municipal finances for urban development	0	0	0	0	0	0	0	0	0	0	0	0	0	0	0	0
10	Skills in the design and execution of municipal projects	-93	-83	-87	-86	-94	-94	-91	-91	-88	-91	-87	-94	-91	-93	-95	-91
11	Development or current state of municipal processes and competencies	-78	-71	-75	-74	-77	-78	-76	-80	-74	-75	-74	-84	-76	-78	-79	-77
12	Level of efficient and equitable coverage of municipal goods and services	-59	-33	-22	-38	-65	-60	-46	-55	-42	-44	-41	-43	-55	-59	-61	-57
	*Average gap in node values in relation to the ideal situation*	-29	-11	-16	-22	-31	-30	-19	-25	-21	-18	-15	-19	-24	-30	-29	-28

The previous results show a decrease (red boxes) in almost all the previously identified gaps, which implies a systemic tendency to converge toward objective values. In particular, one observes a decrease in population needs (UBN) while the efficient and equitable production of municipal goods and services increases insofar as the elements not part of the FCM remain stable. However, it is essential to mention that to reach these results; the system will increase other gaps, such as the need for further use of natural resources to improve municipal finances and a more significant presence of rent-seeking groups in cities.

Notice that even though the gap related to financial dependence on central government transfers decreases, this reduction is marginal compared to the other results, in turn implying the convergence state of FCM calls for reducing the unsatisfied needs of the population and improving municipal performance In tandem, incentives increase to obtain more significant government revenues from the use and sale of natural resources, as well as a desire to expand the presence of power groups in the city administration.

The scenarios presented in the previous table also show that a larger rural population, more involvement in agricultural activities, greater financial dependency on the central government and greater distances from important cities, cause, among other variables, a larger percentage decrease in gaps; nonetheless, this is a decline concerning initial gaps and does not imply a better final scenario compared to the other cases analyzed. This type of municipality improves its performance but during the convergence process, gaps widen for concepts such as the need to attain government revenues through the sale of natural resources, the presence of rent-seeking groups increases and the mayor’s leadership skills to guide urban development decreases.

Up to this point, the strategy has involved introducing an initial state vector *No* given a connection matrix that includes weights *eij* for each cause-effect relationship *Cij* between nodes of the FCM, to reach the final long-term concept values or the convergence state for these values, as shown in Table 7.

**Table 7:** Long-term concept values after using the activation function.

	National level	High EBM	High ISFM	Urban pop.	Rural pop.	Agricult. Act.	Manuf. Ind.	Profess. Act.	Priv. Sect. Employ.	Higher Educat.	Financial Dep.	Univeristies	Seniority	Distance	Altitude	Temperature	Average
N1	0.5	0.5	0.5	0.5	0.5	0.5	0.5	0.5	0.5	0.5	0.5	0.5	0.5	0.5	0.5	0.5	0.5
N2	0.5	0. 6	0. 6	0. 5	0. 5	0. 5	0. 5	0. 5	0. 5	0. 5	0. 5	0. 5	0. 5	0. 5	0. 5	0. 5	0.55
N3	0.6	0.6	0.6	0.6	0.6	0.6	0.6	0.6	0.6	0.6	0.6	0.6	0.6	0.6	0.6	0.6	0.6
N4	0.7	0.7	0.7	0.8	0.7	0.7	0.7	0.7	0.7	0.7	0.8	0.8	0.7	0.7	0.7	0.7	0.7
N5	0.7	0.7	0.7	0.7	0.7	0.7	0.7	0.7	0.7	0.7	0.7	0.7	0.7	0.7	0.7	0.7	0.7
N6	0.6	0.6	0.6	0.6	0.6	0.6	0.6	0.6	0.6	0.6	0.7	0.7	0.6	0.6	0.6	0.6	0.6
N7	0.7	0.7	0.7	0.7	0.7	0.7	0.7	0.7	0.7	0.7	0.7	0.7	0.7	0.7	0.7	0.7	0.7
N8	0.7	0.7	0.7	0.7	0.7	0.7	0.7	0.7	0.7	0.7	0.7	0.7	0.7	0.7	0.7	0.7	0.7
N9	0.9	0.9	0.9	0.9	0.9	0.9	0.9	0.9	0.9	0.9	0.9	0.9	0.9	0.9	0.9	0.9	0.9
N10	0.8	0.8	0.8	0.8	0.8	0.8	0.8	0.8	0.8	0.8	0.8	0.8	0.8	0.8	0.8	0.8	0.8
N11	0.8	0.8	0.8	0.8	0.8	0.8	0.8	0.8	0.8	0.8	0.8	0.8	0.8	0.8	0.8	0.8	0.8
N12	0.9	0.9	0.9	0.9	0.9	0.9	0.9	0.9	0.9	0.9	0.9	0.9	0.9	0.9	0.9	0.9	0.9

As can be seen, the final concept values in the FCM are similar for the fifteen scenarios based on a connection matrix performing iterations using the sigmoid activation function.

The previous table shows that the system described in the FCM for Ecuadorian municipalities should converge when the needs of the urban population are at a "medium" level (*N*_1_ = 0,49), the growth of the educated and professionally trained population (*N*_2_ = 0,55) is at a slightly above "medium" level, the competitive entrepreneurial sector (*N*_3_ = 0,6) is between levels "medium" and "high," with transfers from the central government (*N*_4_ = 0,75) retaining a "high" importance. This also requires that concepts related to the use of natural resources for city development (*N*_5_ = 0,68), the presence of interest groups (*N*_6_ = 0,65), the development of municipal human capital (*N*_7_ = 0,73), and the mayor’s competency and leadership skills, all figure between "medium" and "high" levels.

It is notable that concepts or nodes that address the importance of municipal finances on city development (*N*_9_ = 0,88); the skills to develop and municipal design projects (*N*_10_ = 0,77); the implementation of processes in municipal governments (*N*_11_ = 0,83); and the efficient and equitable coverage of municipal goods and services, are positioned between "high" and "very high" levels.

### The structure needed to reach goals

The following procedure involves using the long-term node values previously described and the initial connection matrix, along with a genetic optimization algorithm, in order to determine values for the matrix that would allow nodes to stabilize at their optimal level over time. The genetic algorithm used is based on Salmeron (2012) and the Matlab code is attached in the S1 File [42].

Table 8 shows new weights in the connection matrix for each causal relationship after using the genetic algorithm, bearing in mind that the convergence state vector or long-term state vector remains stable despite iterations to reach hidden values in the matrix.

**Table 8:** New weights in the connection matrix after applying the genetic algorithm.

	National level	High EBM	High ISFM	Urban pop.	Rural pop.	Agricult. Act.	Manuf. Ind.	Profess. Act.	Priv. Sect. Employ.	Higher Educat.	Financial Dep.	Univeristies	Seniority	Distance	Altitude	Temperature	Average
C(1,2)	-0.6	-0.5	-0.3	-0.4	-0.5	-0.4	-0.6	-0.5	-0.5	-0.6	-0.5	-0.6	-0.4	-0.5	-0.5	-0.4	-0.5
C(1,4)	0.8	0.4	0.3	1.0	0.5	0.8	0.0	0.3	0.2	0.6	0.6	0.9	0.3	0.3	0.3	0.2	0.5
C(1,5)	0.9	0.7	0.6	0.9	0.8	0.7	0.9	0.8	0.7	0.7	0.8	0.8	0.8	0.7	0.7	0.7	0.8
C(2,3)	0.0	0.0	0.5	0.0	0.4	0.0	0.0	0.1	0.0	0.1	0.0	0.0	0.9	0.3	0.3	0.0	0.2
C(2,6)	0.0	0.0	-0.1	0.0	-0.9	0.0	-0.1	0.0	0.0	-0.1	0.0	-0.2	0.0	-0.2	-0.3	0.0	-0.1
C(2,7)	0.5	0.2	0.8	0.0	1.0	0.2	0.4	1.0	0.1	0.4	0.3	0.0	0.2	0.1	0.9	0.0	0.4
C(2,8)	0.8	0.0	0.8	0.1	0.7	0.9	0.9	0.2	0.1	0.0	0.8	0.0	0.8	0.5	0.4	0.3	0.5
C(3,6)	0.0	0.0	-0.1	-0.2	-0.1	-0.4	-0.4	0.0	0.0	-0.1	-0.2	0.0	0.0	-0.1	0.0	0.0	-0.1
C(3,8)	0.1	1.0	0.1	1.0	0.5	0.1	0.1	0.9	0.7	1.0	0.5	0.8	0.2	0.3	1.0	0.5	0.5
C(3,9)	1.0	0.1	0.8	0.0	1.0	0.8	1.0	0.7	0.4	0.2	0.5	0.7	0.6	0.8	1.0	0.1	0.6
C(4,6)	0.0	0.1	0.1	0.1	0.3	0.2	0.5	0.0	0.1	0.3	0.1	0.5	0.3	0.2	0.5	0.0	0.2
C(4,9)	0.9	0.9	1.0	0.9	0.2	0.8	0.1	0.4	1.0	1.0	1.0	0.6	1.0	1.0	0.9	0.9	0.8
C(5,3)	-0.1	0.0	-0.3	0.0	-0.3	0.0	0.0	0.0	0.0	-0.1	0.0	0.0	-0.7	-0.1	-0.1	0.0	-0.1
C(5,4)	0.6	0.9	0.8	0.7	0.7	0.5	1.0	0.8	1.0	0.7	0.8	0.7	1.0	1.0	1.0	0.9	0.8
C(5,6)	0.2	0.2	0.6	0.4	0.7	0.6	0.2	0.4	0.3	0.2	0.5	0.0	0.1	0.2	0.1	0.3	0.3
C(5,9)	0.1	0.8	0.8	0.9	0.7	0.4	1.0	1.0	1.0	0.9	0.4	0.4	0.0	1.00	0.0	0.9	0.6
C(6,7)	0.0	0.0	0.0	-0.2	-0.1	-0.1	-0.2	0.0	-0.1	-0.3	0.0	-0.2	-0.1	0.0	-0.1	0.0	-0.1
C(6,8)	0.0	0.0	0.0	0.0	-0.2	-0.1	0.0	-0.1	0.0	-0.1	-0.2	0.0	0.0	0.0	-0.5	-0.1	-0.1
C(6,10)	0.0	0.0	0.0	0.0	-0.4	0.0	0.0	0.0	0.0	-0.3	-0.1	-0.1	0.0	0.0	-0.1	0.0	-0.1
C(6,11)	0.0	-0.1	-0.3	0.0	-0.6	-0.1	0.0	0.0	0.0	0.0	-0.1	0.0	0.0	0.0	-0.1	-0.1	-0.1
C(7,10)	0.0	0.6	0.3	0.5	0.4	0.1	0.1	0.4	0.9	0.6	0.3	0.8	0.4	0.4	0.5	0.3	0.4
C(7,11)	1.0	0.9	0.3	0.0	1.0	0.3	1.0	0.5	0.5	0.7	0.0	0.0	0.4	0.5	0.7	0.8	0.5
C(7,12)	1.0	0.5	0.8	1.0	0.9	1.0	0.6	0.7	1.0	1.0	0.7	0.1	1.0	1.0	0.6	0.7	0.8
C(8,7)	0.6	0.8	0.4	1.0	0.4	1.0	1.0	0.2	0.8	0.8	0.6	1.0	1.0	1.0	0.3	1.0	0.7
C(8,9)	0.7	0.8	0.3	1.0	1.0	0.8	0.9	0.9	0.8	0.7	1.0	1.0	0.9	0.1	0.8	0.0	0.8
C(8,11)	0.1	0.8	0.8	1.0	0.9	1.0	1.0	0.3	0.8	1.0	0.9	0.8	0.7	0.8	0.1	0.1	0.7
C(9,10)	1.0	0.6	0.9	0.7	1.0	1.0	1.0	0.8	0.6	0.8	1.0	0.5	0.8	0.7	0.8	0.9	0.8
C(9,12)	1.0	1.0	0.9	0.8	0.5	1.0	0.9	0.7	0.4	0.4	0.9	1.0	0.4	0.5	0.7	0.9	0.7
C(10,11)	0.9	0.4	1.0	0.9	0.6	0.6	0.1	1.0	0.6	0.3	1.0	0.7	1.0	0.7	1.0	0.9	0.7
C(12,12)	0.2	1.0	0.7	0.7	0.9	0.2	0.9	0.7	0.9	1.0	0.8	0.9	1.0	0.7	0.9	0.7	0.8
C(12,1)	-0.6	-0.6	-0.6	-07	-0.5	-0.6	-0.5	-0.6	-0.6	-0.7	-0.6	-0.5	-0.6	-0.6	-0.6	-0.5	-0.6

Thus, for example, in relationship *C*(1,2), where the higher the Unsatisfied Basic Needs (UBN), the lower the educated and professionally trained population, we observe that in most municipalities, despite their characteristics, this effect is situated between "low" and "medium" levels.

Relationship *C*(1,4) determines that greater UBN implies higher resource transfers from the central government; this influence is high and very high for urban municipalities, with population working in the agricultural sector, high financial dependency and access to university education. However, this causal relationship is low for scenarios where cities are located far away from important urban centers, have a warm-humid climate, and lie below 1500 m.a.s.l.

The causal relationship for arc *C*(1,5), where UBN positively motivates the use of natural resources for municipal development, is high and very high for all the proposed scenarios. This reveals a national structural problem by highlighting a long-term negative incentive to use natural resources to minimize poverty rates in municipalities.

Relationship *C*(2,3), where a higher educated and professionally trained population implies a more significant presence and development of a competitive entrepreneurial sector, was low for almost all types of municipalities, suggesting that given the proposed FCM, it is not considered fundamental for city development throughout the country. The same happens when considering relationship *C*(2,6), where a higher educated population should reduce the presence of interest groups. The use of the genetic algorithm predicts a low connection in the different scenarios previously described.

Arc *C*(2,7), where the higher the level of educated population in the city, the more human capital included in the municipal government, would show an increased effect on municipalities with an elevated ISFM, towns with a high rural population, a high number of professional activities and a territory with a temperature above 20 degrees Celsius. On average, the influence of this causal relationship is between low and medium.

Relationship *C*(2,8) that determines a larger educated population will positively influence the election of a capable mayor with a forward-looking vision and leadership skills to transform the city, has a significant impact on municipalities with a high ISFM, rural population, agricultural activities, manufacturing industry, lower financial dependency, and cities with over 110 years of being municipal capitals.

Given the new adjacency matrix, the arcs for the following causal relationships would have a minimal effect on the FCM: more development of a competitive entrepreneurial sector lessens the pressure of interest groups *C*(3,6); more significant revenues from natural resources reduce the development of the entrepreneurial sector *C*(5,3); more interest groups reduce municipal human capital *C*(6,7); more interest groups negatively influence the mayor’s abilities, vision for the future and leadership skills *C*(6,8); as well as the municipal ability to design and execute investment projects *C*(6,10); and the municipal ability for process development *C*(6,11).

Relationships *C*(4,6) and *C*(5,6), which determine that the higher the transfers from the central government and the greater the need to exploit natural resources, the higher the presence of interest groups, would thereby display a low influence.

Overall, the node related to the presence of interest groups in the canton would not influence the proposed system or the performance of municipalities.

The following causal relationships would have a medium influence level: a more significant number of businesses in the canton improve the mayor’s competence *C*(3,8). In contrast, greater municipal human capital promotes the development and execution of municipal projects C(7,10).

Relationships on a "medium-high" influence range include those where more competitive business improves municipal finances *C*(3,9); a greater need to use natural resources increases transfers from the central government *C*(5,4); higher municipal human capital increases the provision of municipal goods and services *C*(7,12); greater competencies and forward-looking vision of mayors increase human capital in the municipal government *C*(8,7) and improve municipal finances *C*(8,9), while also facilitating the development of municipal processes *C*(8,11).

In the same range, we can locate relationships *C*(9,10), where improved municipal finances positively influence the municipal ability to design and execute projects and provide goods and services *C*(9,12). This ability highly affects the development of municipal processes *C*(10,11), which leads to an improved production and provision of municipal goods and services *C*(11,12). Therefore, if the provision of municipal goods and services improves, this will negatively influence the UBN of the population over a medium to high range.

The performance of municipalities and the quality of life in Ecuadorian cities depend on institutional variables, such as the educational level of the population, the state of municipal finances, the structure of productive activities, among others. Nonetheless, Ecuadorian municipalities also react to other internal and external factors. For example, on an inner level, the municipality requires the mayor’s aptitude and forward-looking vision to develop the city, demanding qualified human capital and financial resources for project development, which in turn leads to a more efficient and equitable production and provision of public goods and services in cities.

On an external level, Ecuatorian municipalities depend on the initial condition set by Unsatisfied Basic Needs (UBN) of the citizenry; if these are high, there exists a greater probability that the population will be less educated. Furthermore, a municipality with high UBN is likely to receive more frequent transfers from the central government, thus deepening its financial dependence. With limited sources of municipal income, the population and even the local authorities will have more significant incentives to demand more exploitation of non-renewable natural resources, such as oil and other minerals, without considering intergenerational equity criteria.

Among internal factors, developing a competitive entrepreneurial sector is decisive to positively influencing the development and retention of human capital in the city. This, in turn, positively impacts municipal finances since more revenue can be collected. It also increases the possibility of developing new municipal projects, given the improved professional municipal competencies. A municipality with these characteristics will have greater probabilities of achieving sustainable basic municipal competencies over time.

**Fig 2:**
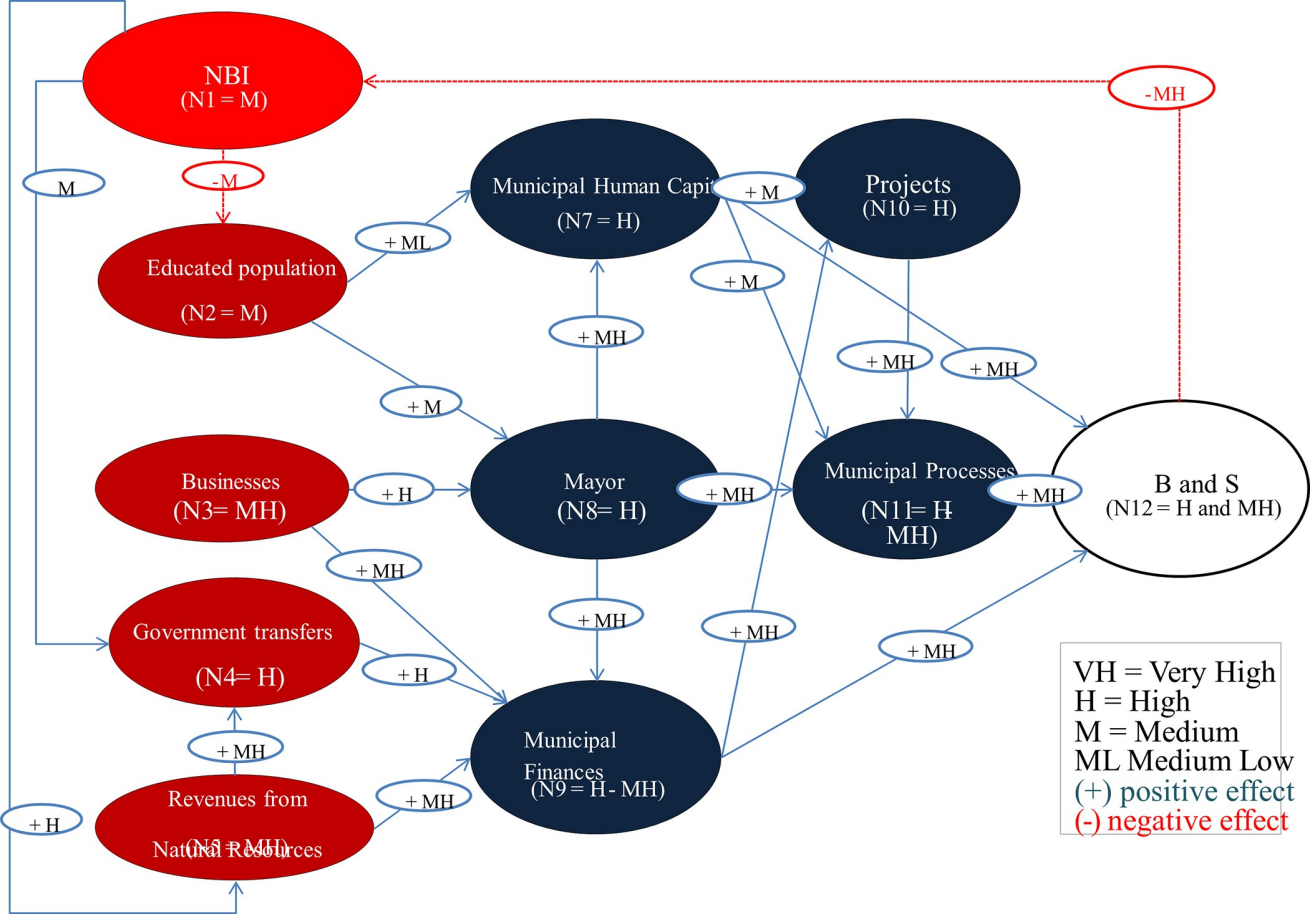
New Fuzzy Cognitive Map after using the genetic algorithm.

Cities are immersed in democratic processes and political dynamics. A city with a low educational level and high UBN is more prone to the appearance of interest groups that seek to gain control and interfere in municipal decisions. Thus, for example, higher revenues from the central government due to the extraction of natural resources can also be a reason for the presence of these interest groups, which negatively influence the mayor’s administration and the technical work carried out by the municipality. This is why it is important to introduce the political system and interest groups as crucial elements that might affect municipal performance (Fig 2).

The FCM reinforces the importance of key concepts in long-term municipal performance, such as the need for a competitive entrepreneurial sector; the improvement of human capital and professional talent within the municipality; having a competent mayor with leadership skills and a forward-looking vision who facilitates the development of municipal projects and processes necessary to achieve an efficient and equitable coverage of goods and services in the city. At the same time, the system developed reveals that less desirable relationships will be sustained, including the dependency on transfers from the central government and the need to exploit natural resources for city development.

The Unsatisfied Basic Needs concept has a medium level of importance, which may be related to the persistence of unequal income distribution, which limits access to a level of consumption of goods and services that can maximize the well-being of the city’s population.

## Conclusions

Integrating the Delphi method, fuzzy cognitive mapping and genetic algorithms have proven beneficial for an in-depth understanding of the determinants of long-term municipal development in Ecuador. Specifically, it has enabled us to address three pivotal questions:

What key concepts describe municipal performance in Ecuador, as measured through the equitable and efficient production and delivery of goods and services?

What are the main constraints faced by municipalities in Ecuador in improving their performance in city management?

Which institutional variables related to public policy design and action plans could exert greater influence on short and long-term municipal performance?

Regarding the first query, Ecuador’s municipal performance is better when the environment includes private sector production, job creation, and purchasing power in the city’s labor market. Evidence also indicates that transfers from the Central Government via tax distribution and non-renewable natural resource sales, such as oil, enable municipalities to develop and execute more public investment projects. Despite fostering financial dependency on the Central Government, these elements boost municipal finances and are preferable if balanced with own resources, such as property taxes or capital expense amortization.

The mayor’s vision of urban development and habitat within the municipality is paramount. Equipping municipal institutions with human capital possessing the requisite capabilities and knowledge to invest in city studies and investment projects is essential, as is developing efficient processes for optimal production and provision of goods and services.

Concerning the second question, some primary constraints municipalities face in improving their performance over time include dependency on transfers from the Ministry of Economy and Finance (taxes and oil), weak private investment attraction, lack of human capital with skills and experience, inefficient tax collection processes, recovery of municipal public works, and absence of feasibility studies for public investment.

As for the third question, in the short term, Ecuadorian municipalities must invest in cadastral information systems to improve city planning, identify potential taxpayer bases, and understand gaps in public goods and services provision. This should be supplemented with municipal programs that educate the population about the importance of reducing tax evasion and avoidance, thereby strengthening municipal finances to increase public goods and services production.

In the medium to long term, mayors need to implement measures to increase the productive base and private job creation in the city, thus generating larger contributions and taxes to the municipality, while facilitating human talent attraction to the city for employment in both the private and municipal public sectors.

Additionally, it has been found that the genetic algorithm helped uncover hidden values in the adjacency matrix to achieve convergence levels of the nodes over time. This process revealed that the concept "presence of rent-seeking groups in the city" does not significantly influence the other nodes in the FCM. This suggests the need to understand better this element identified in meetings with municipal experts.

The results obtained should be taken with caution as they are based on the subjective perceptions of the experts and not on statistical data. Testing the relationships obtained by applying Partial Least Squares Path Modeling (PLS-PM) could be of great interest.

## Supporting information

S1 Raw data Coded survey data prepared for processing using FCM and STATA. (ZIP)

S1 File Fuzzy cognitive maps, Matlab analysis code, list of municipal experts interviewed and survey form (in Spanish). (ZIP)

## References

[1] Chu D., Strand R, Fjelland R. Theories of Complexity. Complexity. 2003;8: 19–30. doi: 10.1002/cplx.10059

[2] Pavard B, Dugdale J. The contribution of complexity theory to the study of socio-technical cooperative systems. In: Minai A, Bar-Yam Y, editors. Unifying themes in complex systems New Research. Springer; 2006. pp. 39–48. Available: http://hal.univ-grenoble-alpes.fr/hal-00952171/

[3] Snowden DJD, Boone MME. A leader’s framework for decision making. Harv Bus Rev. 2007;85: 68. Article 18159787

[4] Cilliers P. Complexity and Postmodernism. Understanding complex systems. 1998. doi: 9786610333837

[5] Blackman T. Complexity theory and the new public management. Soc issues. 2001;1: 1–11. Available: http://www.whb.co.uk/socialissues/tb.htm

[6] Kiel D. Managing chaos and complexity in government: A new paradigm for managing change, innovation, and organizational renewal. Jossey-Bass; 1994. Available: https://www.amazon.es/s/ref=nb_sb_noss?__mk_es_ES=ÅMÅŽÕÑ&url=search-alias%3Denglish-books&field-keywords=Managing+chaos+and+complexity+in+government%3A+A+new+paradigm+for+managing+change%2C+innovation%2C+and+organizational+renewal

[7] Stacey RD. The science of complexity: An alternative perspective for strategic change processes. Strateg Manag J. 1995;16.

[8] Olmedo E. Complexity and chaos in organisations: Complex management. Int J Complex Leadersh Manag. 2010;1: 72–82. doi: 10.1504/IJCLM.2010.035790

[9] Stacey RD. Complexity and Creativity in Organizations. Berrett Koehler; 1996.

[10] Shubik M. Time and Money. The Economy as an Evolving Complex System II. 1997. pp. 263–287.

[11] Rosser JB. On the Complexities of Complex Economic Dynamics. J Econ Perspect. 1999;13: 169–192. doi: 10.1257/jep.13.4.169

[12] Mailath GJ. Do people play Nash equilibrium? Lessons from evolutionary game theory. J Econ Lit. 1998;36: 1347–1374. doi: 10.2307/2564802

[13] Hayek F. The Theory of Complex Phenomena: A Precocious Play on the Epistemology of Complexity. Stud Philos Polit Econ. 1967; 22–42.

[14] Papageorgiou EI, Salmeron JL. A review of fuzzy cognitive maps research during the last decade. IEEE Trans Fuzzy Syst. 2013;21: 66–79. doi: 10.1109/TFUZZ.2012.2201727

[15] Kontogianni AD, Papageorgiou EI, Tourkolias C. How do you perceive environmental change? Fuzzy Cognitive Mapping informing stakeholder analysis for environmental policy making and non-market valuation. Appl Soft Comput J. 2012;12: 3725–3735. doi: 10.1016/j.asoc.2012.05.003

[16] Amirkhani A, Papageorgiou EI, Mohseni A, Mosavi MR. A review of fuzzy cognitive maps in medicine: Taxonomy, methods, and applications. Comput Methods Programs Biomed. 2017;142: 129–145. doi: 10.1016/j.cmpb.2017.02.021 28325441

[17] Tsadiras AK, Margaritis KG. Cognitive mapping and certainty neuron fuzzy cognitive maps. Inf Sci (Ny). 1997;101: 109–130. doi: 10.1016/S0020-0255(97)00001-7

[18] Firmansyah HS, Supangkat SH, Arman AA, Giabbanelli PJ. Identifying the Components and Interrelationships of Smart Cities in Indonesia: Supporting Policymaking via Fuzzy Cognitive Systems. IEEE Access. 2019;7: 46136–46151. doi: 10.1109/ACCESS.2019.2908622

[19] Kafetzis A, McRoberts N, Mouratiadou I. Using fuzzy cognitive maps to support the analysis of stakeholders’ views of water resource use and water quality policy. Stud Fuzziness Soft Comput. 2010;247: 383–402. doi: 10.1007/978-3-642-03220-2_16

[20] Falcone PM, De Rosa SP. Use of fuzzy cognitive maps to develop policy strategies for the optimization of municipal waste management: A case study of the land of fires (Italy). Land use policy. 2020;96: 104680. doi: 10.1016/j.landusepol.2020.104680

[21] Morone P, Yilan G, Imbert E. Using fuzzy cognitive maps to identify better policy strategies to valorize organic waste flows: An Italian case study. J Clean Prod. 2021;319: 128722. doi: 10.1016/j.jclepro.2021.128722

[22] Habib F, Shokoohi A. Classification and Resolving Urban Problems by Means of Fuzzy Approach. Int J Civ Environ Eng. 2009;3: 501–508. Available: http://waset.org/publications/15625/classification-and-resolving-urban-problems-by-means-of-fuzzy-approach

[23] Pluchinotta I, Esposito D, Camarda D. Fuzzy cognitive mapping to support multi-agent decisions in development of urban policymaking. Sustain Cities Soc. 2019;46: 101402. doi: 10.1016/j.scs.2018.12.030

[24] Wildenberg M, Bachhofer M, Adamescu M, De Blust G, Diaz RD, Isak KGQ, et al. Linking thoughts to flows -Fuzzy cognitive mapping as tool for integrated landscape modeling. LandMod 2010: International Conference on Integrative Landscape Modelling. 2010. pp. 1–15. Available: www.symposcience.org

[25] Olazabal M, Pascual U. Use of fuzzy cognitive maps to study urban resilience and transformation. Environ Innov Soc Transitions. 2016;18: 18–40. doi: 10.1016/j.eist.2015.06.006

[26] Axelrod R. Structure of decision: The cognitive maps of political elites. Axelrod R, editor. Princeton University Press; 1976.

[27] Stach W, Kurgan L, Pedrycz W, Reformat M. Genetic learning of fuzzy cognitive maps. Fuzzy Sets Syst. 2005;153: 371–401. doi: 10.1016/j.fss.2005.01.009

[28] Bart Kosko. Neuronal Networks And Fuzzy Systems. A Dynamical Systems Approach to Machine Intelligence. 1992.

[29] Khan MS, Khor S, Chong A. Fuzzy Cognitive Maps With Genetic Algorithm for Goal-Oriented Decision Support. Int J Uncertainty, Fuzziness Knowledge-Based Syst. 2004;12: 31–42. doi: 10.1142/S0218488504003028

[30] Papageorgiou EI, Stylios CD, Groumpos PP. Active Hebbian learning algorithm to train fuzzy cognitive maps. Int J Approx Reason. 2004;37: 219–249. doi: 10.1016/j.ijar.2004.01.001

[31] Dickerson JA, Kosko B. Virtual worlds as fuzzy cognitive maps. Presence: Teleoperators & Virtual Environments. MIT Press; 1994.

[32] Golberg DE. Genetic Algorithms in Search Optimization & Machine Learning. 1989. p. 412. doi: 10.1007/3-540-44673-7

[33] Deb K. Multi-objective Genetic Algorithms: Problem Difficulties and Construction of Test Problems. Evol Comput. 1999;7: 205–230. doi: 10.1162/evco.1999.7.3.205 10491463

[34] Nocedal J, Wright SJ, Robinson SM. Numerical Optimization. 1999.

[35] Herrera F, Lozano M, Verdegay JL. Tackling Real-Coded Genetic Algorithms: Operators and Tools for Behavioural Analysis. Artif Intell Rev. 1998;12: 265–319. doi: 10.1023/A:1006504901164

[36] Michalewicz Z. Genetic Algorithms + Data Structures = Evolution Programs. Computational Statistics & Data Analysis. 1996. pp. 372–373. doi: 10.1007/978-3-662-03315-9

[37] Vázquez Huerga A. A balanced differential learning algorithm in fuzzy cognitive maps. Proc 16th Int Work Qual Reason. 2002; 1–7.

[38] Rescher N. Predicting the Future. Albany, NY: State University of New York; 1998. Available: http://www.sunypress.edu/p-2658-predicting-the-future.aspx

[39] Linstone HA, Turoff M. The Delphi Method—Techniques and Applications. The delphi method—Techniques and applications. 2002. doi: 10.2307/1268751

[40] Stylios CD, Groumpos PP. Fuzzy Cognitive Maps in modeling supervisory control systems. J Intell Fuzzy Syst. 2000;8: 83–98. doi: 10.1016/S0960-0779(98)00303-8

[41] Bueno S, Salmeron JL. Benchmarking main activation functions in fuzzy cognitive maps. Expert Syst Appl. 2009;36: 5221–5229. doi: 10.1016/j.eswa.2008.06.072

[42] Salmeron, Jose L. Fuzzy cognitive maps for artificial emotions forecasting. Applied Soft Computing 12.12 (2012): 3704–3710.

