# Supplementary material for: Fuzzy cognitive maps for municipal governance improvement
Source: PLoS One. 2024 Feb 29;19(2):e0294962. doi: 10.1371/journal.pone.0294962 (PMC10903849; doi:10.1371/journal.pone.0294962)
Supplement: S1 File — (ZIP) [file pone.0294962.s002.zip › Panel of municipal experts.docx]

**Panel of municipal experts**

The 16 municipal experts who contributed to configure the Fuzzy Cognitive Map through the identification of key concepts and the main cause-effect relationships in each of the nodes were the following:

**Lista de expertos municipales**

| **Expert's name**  (a) | **Institution** | Position |
| --- | --- | --- |
| Mauricio Velásquez | Municipality of Guayaquil / CAF | Former Director of Environment of the Municipality of Guayaquil / Climate Change Executive of CAF |
| Sandra Fierro | Ministry of Finance  / CAF | Former Director of Public Credit of the Ministry of Finance / Public Sector Executive |
| Verónica Lojan | Oderbrecht | Former Public Relations Director / former consultant with several Municipalities |
| Mª. Dolores Almeida | UN Women / Ministry of Finance / GIZ | Consultant / Former Deputy Minister of Finance / former GIZ local and municipal development officer |
| Verónica Guzmán | Association of Municipalities of Ecuador (AME) | Director of AME Cooperation |
| Juan Salgado | Association of Municipalities of Ecuador (AME) | Directo ofr Avalúos AME |
| Grace Guerrero | Researcher / Consultant | Professor at the Catholic University of Ecuador / Consultant in local development issues. |
| Diego Guanoluisa | Association of Municipalities of Ecuador (AME) | Appraisal and Cadastre Expert |
| Auki Tituaña | Municipality of Cotacachi | Former Mayor of Cotacachi |
| Andrés Martínez | University of Cuenca | Research Director, expert in water issues in the territories. |
| Andrea Yánez | Universidad de las Américas | Investigadora y Consultora con Gobiernos Municipales |
| Cecilia Lincango | Grupo Faro | Directora de Fortalecimiento Institucional a Gobiernos Autónomos Descentralizados |
| Reydi Jaramillo | Junta Parroquial de Catamayo | Vicepresidente Junta Parroquial de Catamayo |
| Orazio Bellettini | Grupo Faro | Director Ejecutivo |
| Andrea Villarreal | Ministerio de Finanzas | Exdirectora de Relaciones Fiscales con organismos Subnacionales |
| Andrés Cisneros | Association of Municipalities of Ecuador (AME) | Coordinador |

Source: own elaboration.
