# Supplementary material for: Fuzzy cognitive maps for municipal governance improvement
Source: PLoS One. 2024 Feb 29;19(2):e0294962. doi: 10.1371/journal.pone.0294962 (PMC10903849; doi:10.1371/journal.pone.0294962)
Supplement: S1 File — (ZIP) [file pone.0294962.s002.zip › Survey (Spanish).docx]

# Cuestionario aplicado a expertos municipales
